# Supplementary material for: Use of the Polo-like kinase 4 (PLK4) inhibitor centrinone to investigate intracellular signalling networks using SILAC-based phosphoproteomics
Source: Biochem J. 2020 Jul 2;477(13):2451–75. doi: 10.1042/BCJ20200309 (PMC7338032; doi:10.1042/BCJ20200309)
Supplement: Supplementary Figures S1-S7 [file BCJ-477-2451-s1.pdf]

SUPPLEMENTARY TABLES

Supplementary Table 1. Proteome changes induced by centrinone.

List of the identified proteins in the FLAG-WT PLK4 (sheet 1) or the FLAG-G95R PLK4 (sheet 2) cell line and the fold change (FC) in response to centrinone treatment. Statistical analysis was performed using the LIMMA package in R. p-value and adjusted p-values calculated using the Benjamini-Hochberg correction for multiple testing) are detailed for each protein family.

Supplementary Table 2. Phosphoproteome changes induced by centrinone.

List of the identified phosphopeptides, the sites within the proteins and the PTM-score computed site localisation confidence in the FLAG-WT PLK4 (sheet 1) or the FLAG-G95R PLK4 (sheet 2) cell line, and the fold change (FC) in response to centrinone treatment. Statistical analysis was performed using the LIMMA package in R. p-value and adjusted p-values calculated using the Benjamini-Hochberg correction for multiple testing) are detailed for each protein family. Also detailed are the site conservation across 100 species (listed in sheet 3) and the prevlance of prior observation in either PhosphositePlus (PSP) or Peptide Atlas (PA) – see Methods for detailed information.

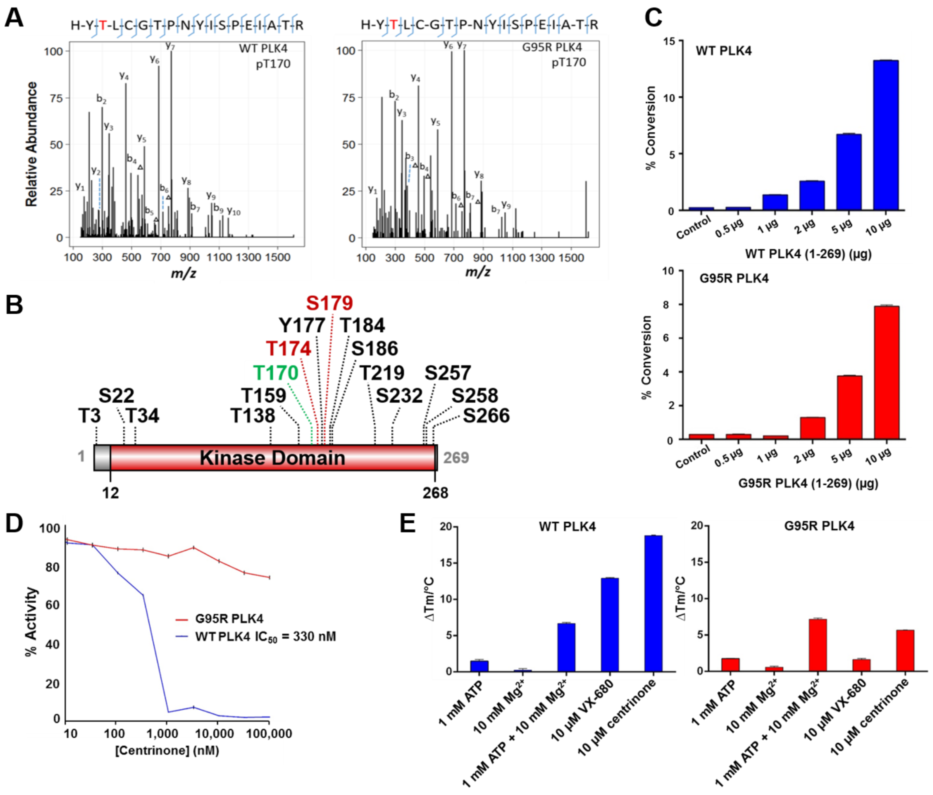

SUPPLEMENTARY FIGURES

Supplementary Figure 1. Recombinant WT and G95R PLK4 are catalytically active, autophosphorylating on multiple residues, and G95R PLK4 is highly resistant to inhibition with centrinone.

(**A**) Purified recombinant WT or G95R PLK4 (1-269) were digested with trypsin and analysed by LC-MS/MS. MS2 spectra generated by HCD shows expression-induced autophosphorylation at T170 (a known site of phosphorylation required for activity) within the PLK4 activation loop in both recombinant WT & G95R PLK4. (**B**) Autophosphorylation sites identified in WT and G95R PLK4 (1-269) are depicted. T170 is shown in green. Phosphosites in red (T174, S179) conform to a [pS/pT]P consensus. (**C**) Purified recombinant WT or G95R human PLK4 (1–269) were assayed with a fluorescent PLK4 peptide substrate (5'-FAM-FLAKSFGSPNRAYKK) in the presence of 1 mM ATP. (**D**) Purified recombinant WT (blue) or G95R (red) PLK4 (1-269) were incubated with fluorescent peptide substrate in the presence of DMSO (control) or the indicated concentrations of centrinone and 1 mM ATP. The extent of peptide phosphorylation (converted to activity) was analysed by mobility shift assay using the EZ Reader platform. Data in (**C**) are from triplicate assays performed twice. Data in (**D**) are from a single triplicate assay. Similar results were seen in a

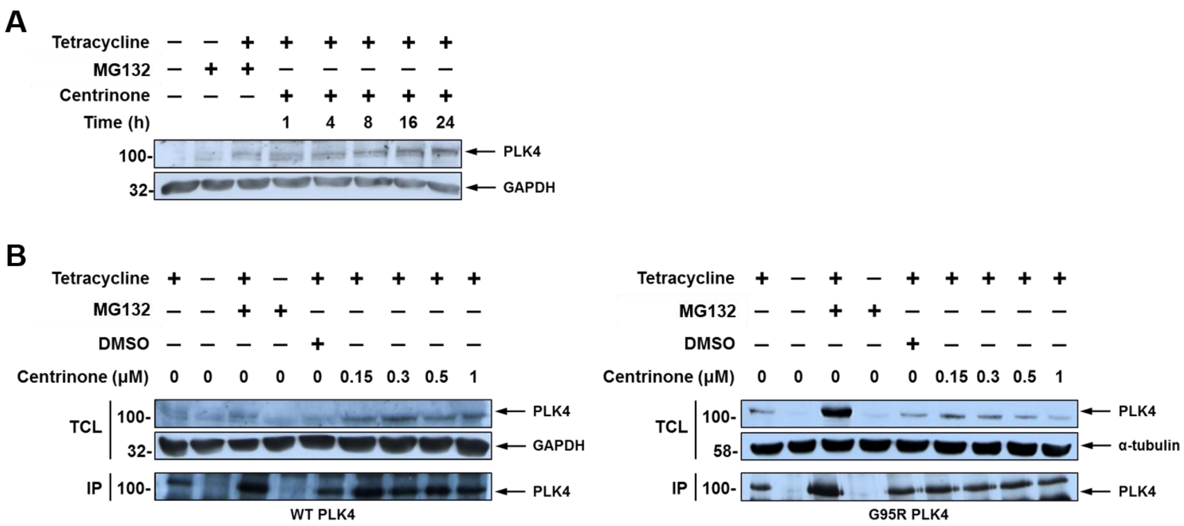

separate experiment. (**E**) DSF analysis of WT (blue) or G95R (red) PLK4 in the presence of the indicated concentrations of nucleotides, metals or inhibitor compounds.

Supplementary Figure 2. FLAG-WT PLK4 but not FLAG-G95R PLK4 is stabilised by centrinone in a concentration-dependent manner.

Expression of FLAG-WT PLK4 or FLAG-G95R PLK4 was induced with 1 µg/mL tetracycline for 18 hours. Cells were incubated with 10 µM MG-132 for 4 hours and either (**A**) 150 nM centrinone for the times indicated, or (**B**) with the indicated concentrations of centrinone for 4 hours. Total cell lysates (TCL) and immunoprecipitated FLAG-PLK4 were analysed by western blotting using the indicated antibodies.

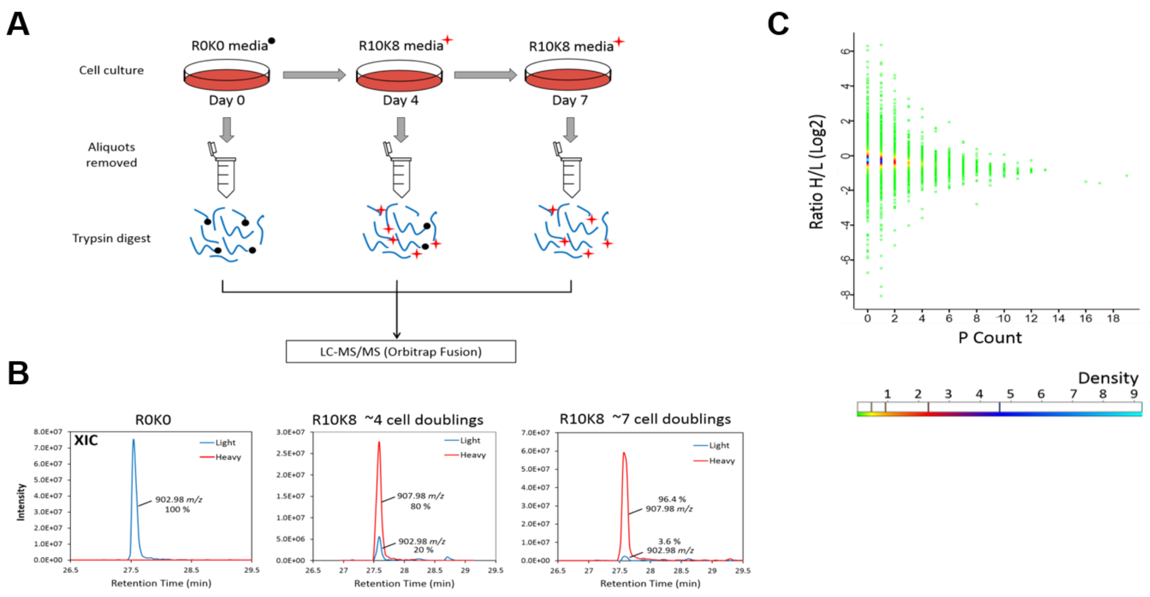

Supplementary Figure 3. Efficient SILAC labelling of U2OS-FLAG WT PLK4 cells.

(**A**) U2OS cells cultured in ‘light’ media were sub-cultured into ‘heavy’ media (R10K8) containing the stable isotopes arginine ($^{13}C_6$ $^{15}N_4$) and lysine ($^{13}C_6$ $^{15}N_2$), allowing incorporation of the isotope-labelled amino acids into newly synthesised proteins during cell growth and protein turnover. At each passage, an aliquot of cells were removed for analysis by LC-MS/MS following tryptic proteolysis, to assess incorporation of the heavy labelled amino acids. (**B**) The extracted ion chromatograms (XIC) show the ion signals for an exemplar doubly charged unlabelled tryptic peptide ion at m/z 902.98 unlabelled. (Left) XIC of the light (unlabelled) peptide in blue, with increasing amount of ‘heavy’ labelled peptide ion (m/z 907.98; 10 Da mass difference) being observed after ~4 and then 7 cell doublings (red), at which point labelling has reached over 96%. (**C**) A density plot was generated to assess the metabolic conversion of Arg to Pro. Non-normalised H/L ratios were plotted against the total proline count from all identified peptides. The data points are colour coded based on density. No global drift

toward the unlabelled peptides were observed, confirming no significant metabolic conversion.

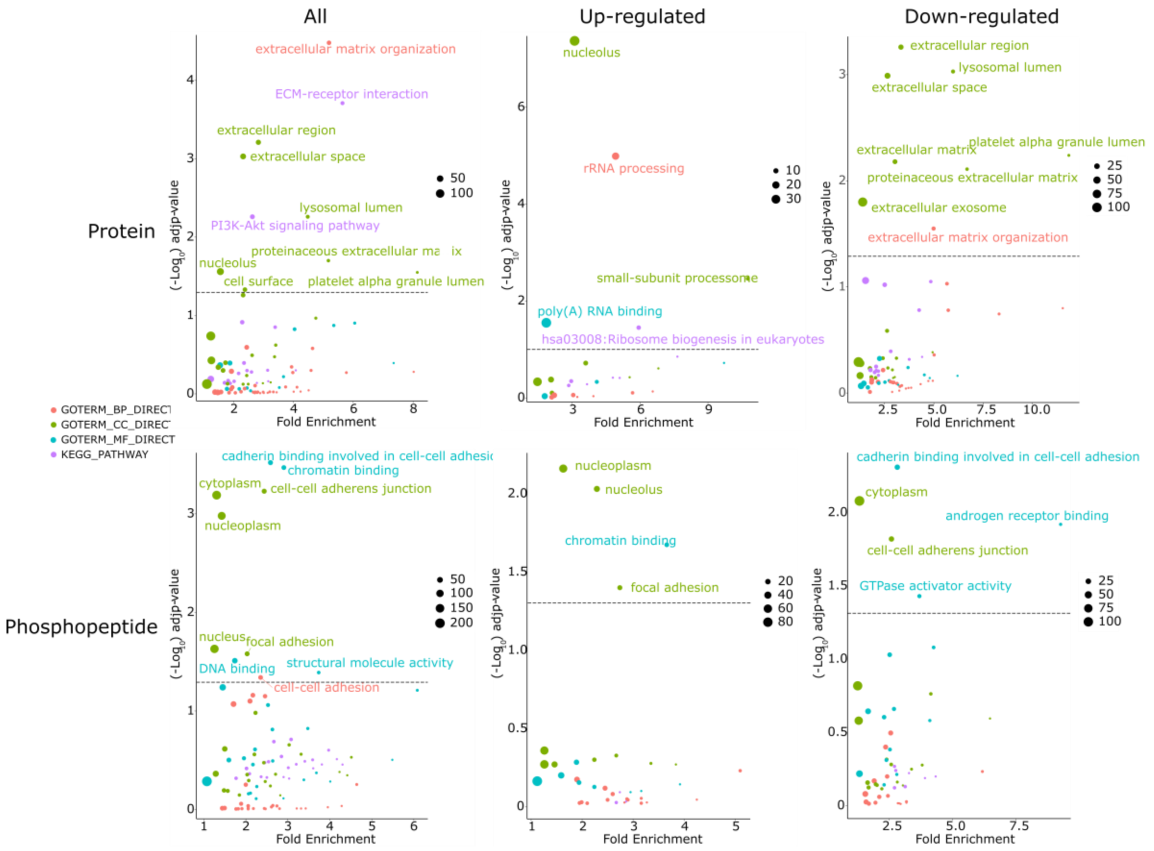

Supplementary Figure 4. GO term enrichment analysis.

Data for significantly regulated protein expression (top), or proteins with differentially regulated phosphopeptides (bottom) observed after centrinone treatment of FLAG-WT PLK4 U2OS cells. Proteins/phosphopeptides with a Benjamini-Hochberg adjusted p-value ≤0.05 are labelled. BP = biological process (red); CC = cellular compartment (green); MF = molecular function (cyan); KEGG pathway (purple). The size of the node is representative of the number of proteins contributing to a select category.

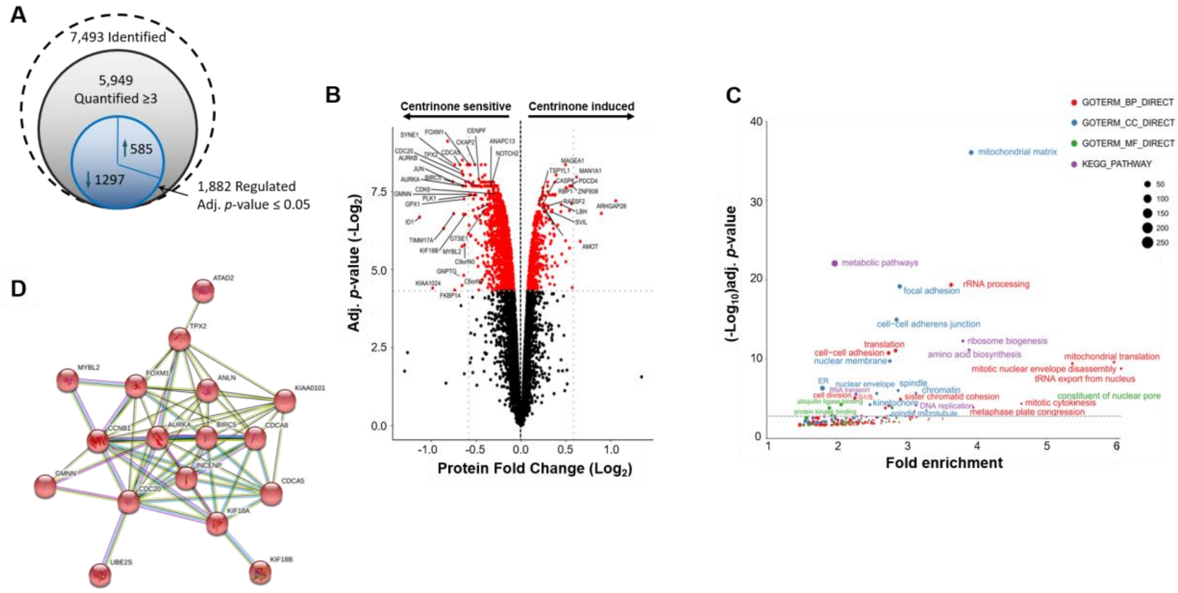

Supplementary Figure 5. Significant protein regulation is observed in FLAG-G95R PLK4 cells following centrinone treatment.

(**A**) Total numbers of identified, quantified and differentially regulated proteins are indicated. (**B**) Volcano plot showing protein fold changes following Bayesian statistical analysis to evaluate significant differences. Log_2-fold change (Heavy/Light) are presented as a function of the $–Log_2$ Benjamini-Hochberg adjusted p-value; those proteins with an adjusted p-value ≤0.05 are highlighted in red. Select data points are annotated with their protein accession number. (**C**) GO term enrichment analysis of significantly regulated proteins using DAVID. Proteins/phosphopeptides with a Benjamini-Hochberg adjusted p-value ≤0.05 are labelled. BP = biological process (red); CC = cellular compartment (blue); MF = molecular function (green); KEGG pathways (purple). (**D**) STRING interaction analysis reveals a network of down-regulated mitotic proteins.

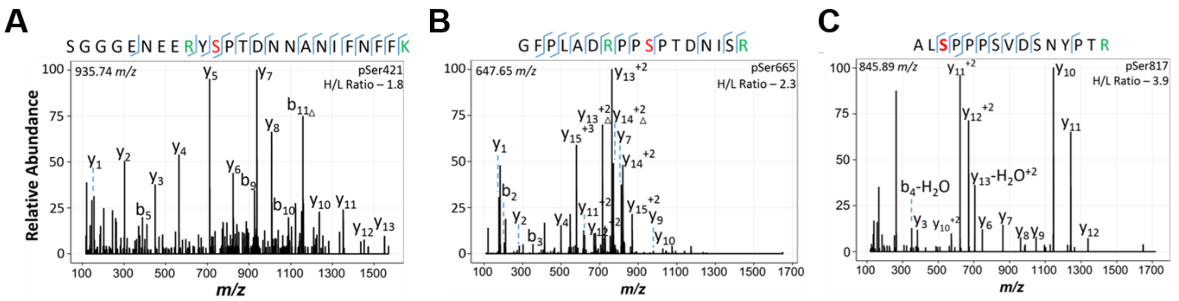

Supplementary Figure 6. Centrinone-mediate upregulation of PLK4 phosphosites. Three significantly up-regulated (adj. p value ≤0.05) PLK4 phosphosites were identified from U2OS FLAG-WT PLK4 cells following treatment with centrinone (300 nM centrinone for 4 hours). The peptide sequence and the identified phosphosite (red) is detailed on each tandem mass spectrum. SILAC labelled K/R residues are in green. (**A**) Doubly charged ion at m/z 935.74, identifying pSer421 as the site. (**B**) Doubly charged ion at m/z 647.65, identifying pSer665. (**C**) Doubly charged ion at m/x 845.89, identifying pSer817 as the site.

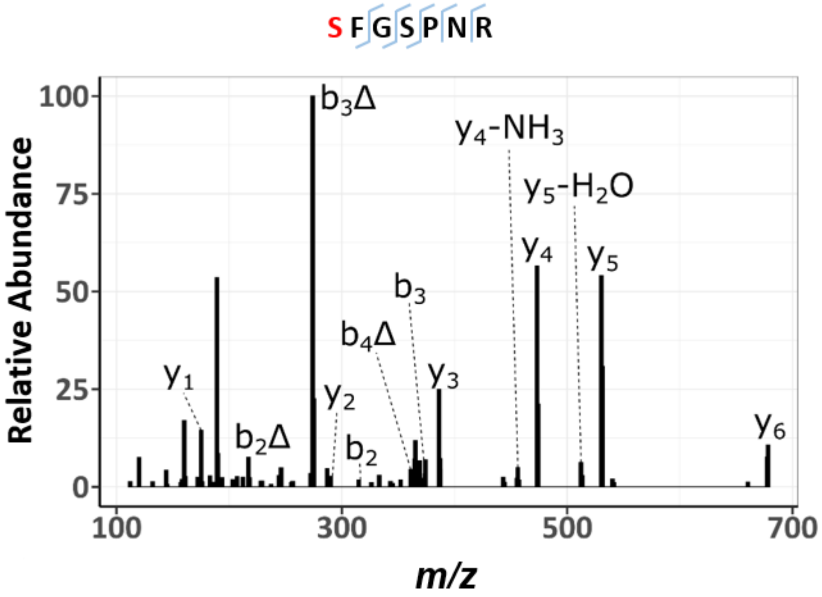

Supplementary Figure 7. HCD product ion mass spectrum of the digested FLAKSFGSPNRAYKK peptide substrate.

Fluorescently-labelled FLAKSFGSPNRAYKK peptide was incubated with PLK4 in 25 mM HEPES (pH 7.4), 1 mM ATP, 5 mM $MgCl_2$, and 0.001% (v/v) Brij 35 at 37°C. Reactions were then digested with trypsin to remove the fluorescent tag, and the digest subjected to LC-MS/MS analysis. Note the digested peptide yields the sequence SFGSPNR with a precursor m/z value of 422.6711. Red denotes the site of phosphorylation. Δ indicates H_3PO_4 loss.
